# Supplementary material for: Mild replication stress causes premature centriole disengagement via a sub-critical Plk1 activity under the control of ATR-Chk1
Source: Nat Commun. 2023 Sep 29;14:6088. doi: 10.1038/s41467-023-41753-1 (PMC10541884; doi:10.1038/s41467-023-41753-1)
Supplement: Supplementary file 3 — Description of Additional Supplementary Files [file 41467_2023_41753_MOESM3_ESM.pdf]

Description of Additional Supplementary Files

File name: Supplementary Movie 1

Description: Live-cell imaging of hTERT-RPE1 stably expressing EB3-GFP and H2B-mCherry and treated with DMSO control. Every image was captured at an interval of 3 m. The movie is shown at 5 frames per second (fps). Scale bars, 5 µm.

File name: Supplementary Movie 2

Description: Live-cell imaging of hTERT-RPE1 stably expressing EB3-GFP and H2B-mCherry and treated with Aphidicolin. Every image was captured at an interval of 3 m. The movie is shown at 5 frames per second (fps). Scale bars, 5 µm.

File name: Supplementary Movie 3

Description: Live-cell imaging of hTERT-RPE1 stably expressing EB3-GFP and H2B-mCherry and treated with Wee1i-1. Every image was captured at an interval of 3 m. The movie is shown at 5 frames per second (fps). Scale bars, 5 µm.

File name: Supplementary Movie 4

Description: Live-cell imaging of hTERT-RPE1 stably expressing EB3-GFP and H2B-mCherry and treated with Wee1i-2. Every image was captured at an interval of 3 m. The movie is shown at 5 frames per second (fps). Scale bars, 5 µm.

File name: Supplementary Movie 5

Description: Live-cell imaging of hTERT-RPE1 stably expressing EB3-GFP and H2B-mCherry and treated with Aphidicolin and Wee1i-1. Every image was captured at an interval of 3 m. The movie is shown at 5 frames per second (fps). Scale bars, 5 µm.

File name: Supplementary Movie 6

Description: Live-cell imaging of hTERT-RPE1 stably expressing EB3-GFP and H2B-mCherry and treated with Aphidicolin and Wee1i-2. Every image was captured at an interval of 3 m. The movie is shown at 5 frames per second (fps). Scale bars, 5 µm.
