# Supplementary material for: Mild replication stress causes premature centriole disengagement via a sub-critical Plk1 activity under the control of ATR-Chk1
Source: Nat Commun. 2023 Sep 29;14:6088. doi: 10.1038/s41467-023-41753-1 (PMC10541884; doi:10.1038/s41467-023-41753-1)
Supplement: Supplementary file 10 — Reporting Summary [file 41467_2023_41753_MOESM10_ESM.pdf]

nature portfolio

Corresponding author(s): Patrick Meraldi

Last updated by author(s): Aug 31, 2023

Reporting Summary

Nature Portfolio wishes to improve the reproducibility of the work that we publish. This form provides structure for consistency and transparency in reporting. For further information on Nature Portfolio policies, see our Editorial Policies and the Editorial Policy Checklist.

Statistics

For all statistical analyses, confirm that the following items are present in the figure legend, table legend, main text, or Methods section.

n/a	Confirmed	
☐	☒	The exact sample size (*n*) for each experimental group/condition, given as a discrete number and unit of measurement
☐	☒	A statement on whether measurements were taken from distinct samples or whether the same sample was measured repeatedly
☐	☒	The statistical test(s) used AND whether they are one- or two-sided<br>*Only common tests should be described solely by name; describe more complex techniques in the Methods section.*
☒	☐	A description of all covariates tested
☐	☒	A description of any assumptions or corrections, such as tests of normality and adjustment for multiple comparisons
☐	☒	A full description of the statistical parameters including central tendency (e.g. means) or other basic estimates (e.g. regression coefficient) AND variation (e.g. standard deviation) or associated estimates of uncertainty (e.g. confidence intervals)
☐	☒	For null hypothesis testing, the test statistic (e.g. *F*, *t*, *r*) with confidence intervals, effect sizes, degrees of freedom and *P* value noted<br>*Give P values as exact values whenever suitable.*
☒	☐	For Bayesian analysis, information on the choice of priors and Markov chain Monte Carlo settings
☒	☐	For hierarchical and complex designs, identification of the appropriate level for tests and full reporting of outcomes
☒	☐	Estimates of effect sizes (e.g. Cohen's *d*, Pearson's *r*), indicating how they were calculated

Our web collection on statistics for biologists contains articles on many of the points above.

Software and code

Policy information about availability of computer code

Data collection	For FRET analysis Dual channel images were acquired on Spinning disk microscope (Nipkow disk) with Visiview 4.00.10 software (Visitron)<br>Expansion microscopy images were acquired on Deltavision microscope with Softworx 6.5.2 (GE Healthcare) software.<br>Immunoflourescence images were acquired on Deltavision microscope with Softworx 6.5.2 (GE Healthcare) software.<br>Live cell imaging data was acquired on Nikon Ti Microscope using NIS Elements 4.30.02 software (Nikon).<br>STED images were acquired on TCS SP8 STED microscope (Leica Microsystems) using LAS-X 1.4.5 Imaging software (Leica Microsystems).
Data analysis	All the Immuno-fluorescence images (IF, STED, U-ExM, and FRET) were analyzed in ImageJ/Fiji.<br>Statistical data were analyzed with Graphpad Prism 9 (Graphpad)

For manuscripts utilizing custom algorithms or software that are central to the research but not yet described in published literature, software must be made available to editors and reviewers. We strongly encourage code deposition in a community repository (e.g. GitHub). See the Nature Portfolio guidelines for submitting code & software for further information.

Data

Policy information about availability of data

All manuscripts must include a data availability statement. This statement should provide the following information, where applicable:

- Accession codes, unique identifiers, or web links for publicly available datasets
- A description of any restrictions on data availability
- For clinical datasets or third party data, please ensure that the statement adheres to our policy

Source data are provided with this paper. All the Raw data related to figures and supplementary figures (representative images and movies) will be available on: https://doi.org/10.26037/yareta:4f2f6xydgjfmvc6alqq6fbv2aq
Due to the large size (> 4TB), all raw live cell movie data used for analyses will be made available on request by sending external hard disks.

Human research participants

Policy information about studies involving human research participants and Sex and Gender in Research.

Reporting on sex and gender	N/A
Population characteristics	N/A
Recruitment	N/A
Ethics oversight	N/A

Note that full information on the approval of the study protocol must also be provided in the manuscript.

Field-specific reporting

Please select the one below that is the best fit for your research. If you are not sure, read the appropriate sections before making your selection.

☒ Life sciences ☐ Behavioural & social sciences ☐ Ecological, evolutionary & environmental sciences

For a reference copy of the document with all sections, see nature.com/documents/nr-reporting-summary-flat.pdf

Life sciences study design

All studies must disclose on these points even when the disclosure is negative.

Sample size	Sample size and number of biological repeats for each experiment are indicated in all figure legends or in the "Statistics and reproducibility" section. In all the experiments, at least 3 independent biological repeats were performed. In each case the sample size was chosen to fit the statistical test used for testing the null hypothesis.
Data exclusions	No data was excluded from the analysis.
Replication	All quantifiable experiments were replicated in at least 3 independent experiments (sometimes more than 3) to ensure the reproducibility of the results
Randomization	Our study is based solely on basic research. Moreover, due to the variations within the same condition and the morphological modifications following the different treatments used which would inevitably give us an indication of the condition analyzed, makes the randomization not applicable to our study. Therefore, no formal randomization techniques were used.<br>However, we performed all of our experiments as consistently as possible and most of our measurements are based on a large number of analyzed conditions.
Blinding	Blinding was not necessary during data collection (Image acquisition) as information regarding centriole disengagement or fluorescence intensity can only be obtained during Image analysis. All the quantifiable parameters were extracted from the images using automated image analysis softwares that excluded experimentator-induced biases, further rendering the usage of blinding approaches unnecessary. Furthermore, our study does not work with human subjects/patients/animals.

Reporting for specific materials, systems and methods

We require information from authors about some types of materials, experimental systems and methods used in many studies. Here, indicate whether each material, system or method listed is relevant to your study. If you are not sure if a list item applies to your research, read the appropriate section before selecting a response.

Materials & experimental systems

n/a	Involved in the study
☐	☒ Antibodies
☐	☒ Eukaryotic cell lines
☒	☐ Palaeontology and archaeology
☒	☐ Animals and other organisms
☒	☐ Clinical data
☒	☐ Dual use research of concern

Methods

n/a	Involved in the study
☒	☐ ChIP-seq
☒	☐ Flow cytometry
☒	☐ MRI-based neuroimaging

Antibodies

Antibodies used

- - Primary Antibodies:
Mouse anti-α-tubulin (Geneva antibody facility: AA345-M2a; 1:250: ExM)
Mouse anti-β-tubulin (Geneva antibody facility: AA344-M2a; 1:250: ExM)
Mouse anti-α-tubulin (Clone DM1α; Sigma Aldrich, T9026, 1:5000: Western Blotting)
Rabbit polyclonal anti-Pericentrin (abcam: ab4448; 1:250: ExM, 1:1000: STED; 1:2000: Immunoflourescence)
Rabbit polyclonal anti-CEP57 (GeneTex: GTX115931; 1:250: ExM)
Mouse anti-Cyclin-A2 (Clone: E23.1, abcam: ab38; 1:1000: Western Blotting)
Mouse anti-Cyclin-B1 (Clone V152 abcam: ab72; 1:1000: Western Blotting)
Mouse anti-CENP-F (Clone: 14C10 /1D8; abcam: ab90; 1:1000: STED)
Mouse anti-Separase (abcam: ab16170; 1:1000: Western Blotting)
Rabbit anti-Centrobin (Sigma Aldrich HPA023321; 1:250: ExM)
Mouse anti-c-Myc (Thermo Scientific: MA1-980; 1:1000: Immunoflourescence)

- - - Alexa Fluor–conjugated secondary antibodies in Immunofluorescence/U-ExM– Dilution 1:500:
Donkey anti-Mouse IgG (H+L) Alexa Fluor 488 (Invitrogen; A-21202)
Goat anti-Rabbit IgG (H+L) Alexa Fluor 488 (Invitrogen; A-11008)
Goat anti-Rabbit IgG (H+L) Alexa Fluor 568 (Invitrogen; A-11036)
Goat anti-Mouse IgG (H+L) Alexa Fluor 568 (Invitrogen; A-11031)

- - HRP-Conjugated secondary antibody in Western Blotting - Dilution 1:10,000
Goat anti-Mouse antibody for western blotting (Thermo Fisher: Cat # 32430)

- - STAR RED Congugated secondary antibody used in STED imaging - Dilution: 1:1,000
Goat anti-Rabbit STAR RED antibody (Abberior instruments GmbH: Cat # 2-0002-011-9)

Validation

- Mouse anti-α-tubulin (Geneva antibody facility: AA345-M2a; 1:250: ExM) was previously tested and validated in the manuscript (doi:10.24450/journals/abrep.2019.e17)
- Mouse anti-β-tubulin (Geneva antibody facility: AA345-M2a; 1:250: ExM) was previously tested and validated in the manuscript (doi:10.24450/journals/abrep.2019.e17)
- Mouse anti-α-tubulin (Clone DM1α; Sigma Aldrich, T9026, 1:5000: Western Blotting) was previously validated by the manufacturer (https://www.sigmaaldrich.com/CH/en/product/sigma/t9026)
-Rabbit polyclonal anti-Pericentrin (abcam: ab4448; 1:250: ExM, 1:1000: STED) was previously validated by the manufacturer (https://www.abcam.com/pericentrin-antibody-centrosome-marker-ab4448.html)
- Rabbit polyclonal anti-CEP57 (GeneTex: GTX115931; 1:250: ExM) was previously validated by the manufacturer (https://www.genetex.com/Product/Detail/CEP57-antibody-N1C1/GTX115931)
- Mouse anti-Cyclin-A2 (Clone: E23.1, abcam: ab38; 1:1000: Western Blotting) was previously validated by manufacturer (https://www.abcam.com/cyclin-a2-antibody-e231-ab38.html)
- Mouse anti-Cyclin-B1 (Clone V152, abcam: ab72; 1:1000: Western Blotting) was previously validated by the manufacturer (https://www.abcam.com/cyclin-b1-antibody-v152-ab72.html)
- Mouse anti-CENP-F (Clone: 14C10 /1D8; abcam: ab90; 1:1000: STED) was previously validated by the manufacturer (https://www.abcam.com/cenpf-antibody-14c10-1d8-ab90.html)
Mouse anti-Separase (abcam: ab16170; 1:1000: Western Blotting) was previously validated by the manufacturer (https://www.abcam.com/products/primary-antibodies/separase-antibody-xj11-1b12-ab16170.html)
Rabbit anti-Centrobin (Sigma Aldrich HPA023321; 1:250: ExM) was previously validated by the manufacturer (https://www.sigmaaldrich.com/CH/en/product/sigma/hpa023321)
Mouse anti-c-Myc (Thermo Scientific: MA1-980; 1:1000: Immunoflourescence) was previously validated by the manufacturer (https://www.thermofisher.com/antibody/product/c-Myc-Antibody-clone-9E10-Monoclonal/MA1-980)

Eukaryotic cell lines

Policy information about [cell lines and Sex and Gender in Research](#)

Cell line source(s)

- hTERT-RPE1 cells were obtained from ATCC (ATCC; CRL-4000, CCL-2).
- hTERT-RPE1 EB3-eGFP/H2B-mCherry cell-line was kindly provided by Willy Krek, ETH Zurich, Switzerland and has been used in the previous study from the lab in Whlhelm et al., 2019 (https://www.nature.com/articles/s41467-019-11584-0#Sec21).
- hTERT-RPE1 Myc-OSTR1 cell-line was kindly provided by Helfrid Hochegger, University of Sussex, United Kingdom and was described for the first time in Hégarat et al., 2020 (https://www.embopress.org/doi/full/10.15252/embj.2020104419)
- hTERT-RPE1 Cyclin-A2-double degron cell-line was kindly provided by Helfrid Hochegger, University of Sussex, United Kingdom and was described for the first time in Hégarat et al., 2020 (https://www.embopress.org/doi/full/10.15252/

	embj.2020104419)<br>- hTERT-RPE1 Cyclin-B1-double degron cell-line was kindly provided by Helfrid Hochegger, University of Sussex, United Kingdom and was described for the first time in Hégarat et al., 2020 (https://www.embopress.org/doi/full/10.15252/embj.2020104419)<br>- hTERT-RPE1 Plk1 FRET Sensor cell-line was generated in the lab for this study and the process is described in the materials and methods section.<br>- hTERT-RPE1 Myc-Separase cell-line was generated in the lab for this study and the process is described in the materials and methods section.
Authentication	None of the cell lines was authenticated
Mycoplasma contamination	All cell lines are tested for mycoplasma every 6 months and are negative for any mycoplasma contamination.
Commonly misidentified lines<br>(See ICLAC register)	All the cell lines used in this study are not included in the list of known misidentified cell lines from the International Cell Line Authentication Committee.
