# Supplementary figures and images for: Mild replication stress causes premature centriole disengagement via a sub-critical Plk1 activity under the control of ATR-Chk1
Source: Nat Commun. 2023 Sep 29;14:6088. doi: 10.1038/s41467-023-41753-1 (PMC10541884; doi:10.1038/s41467-023-41753-1)

Fig S2B

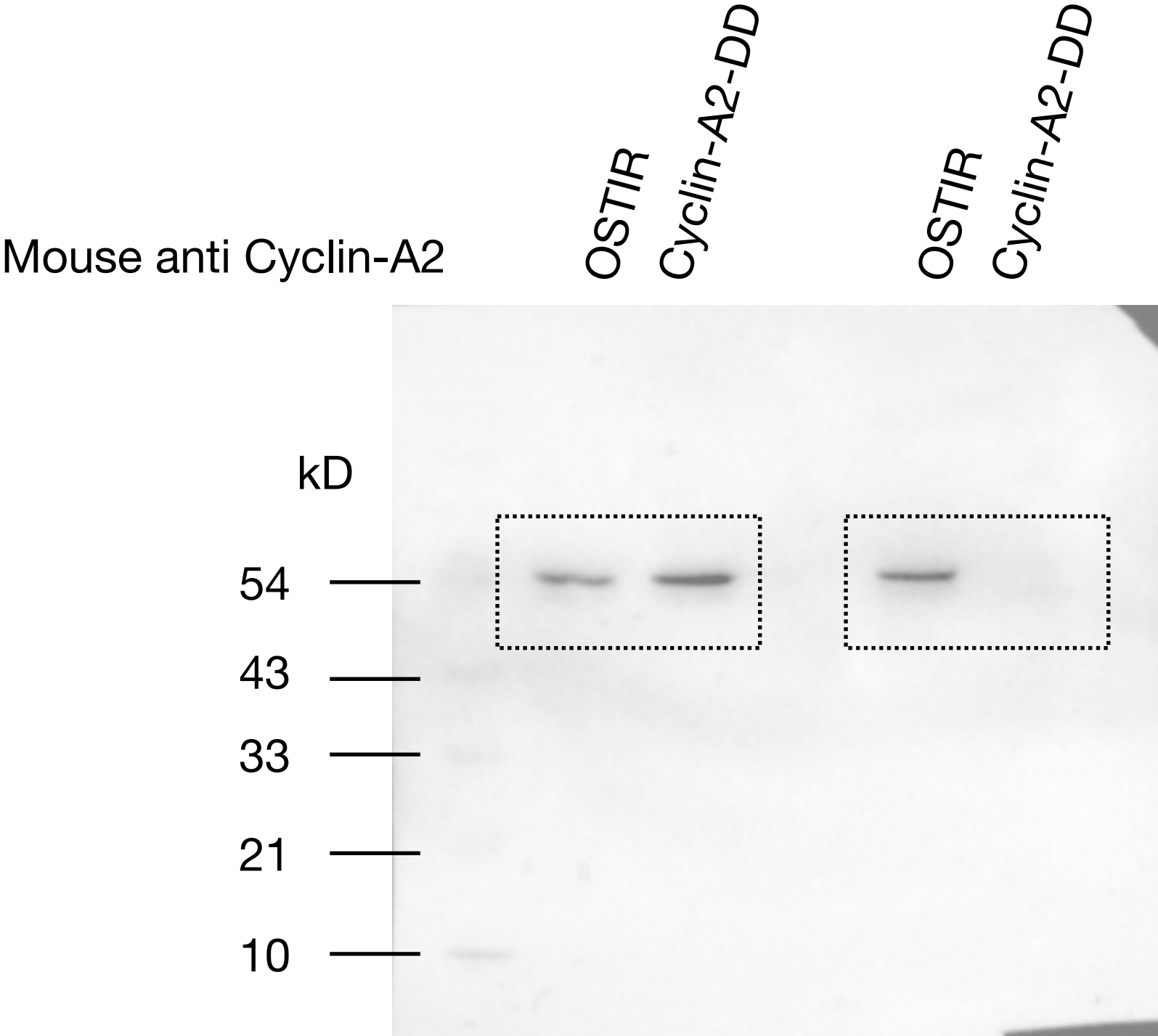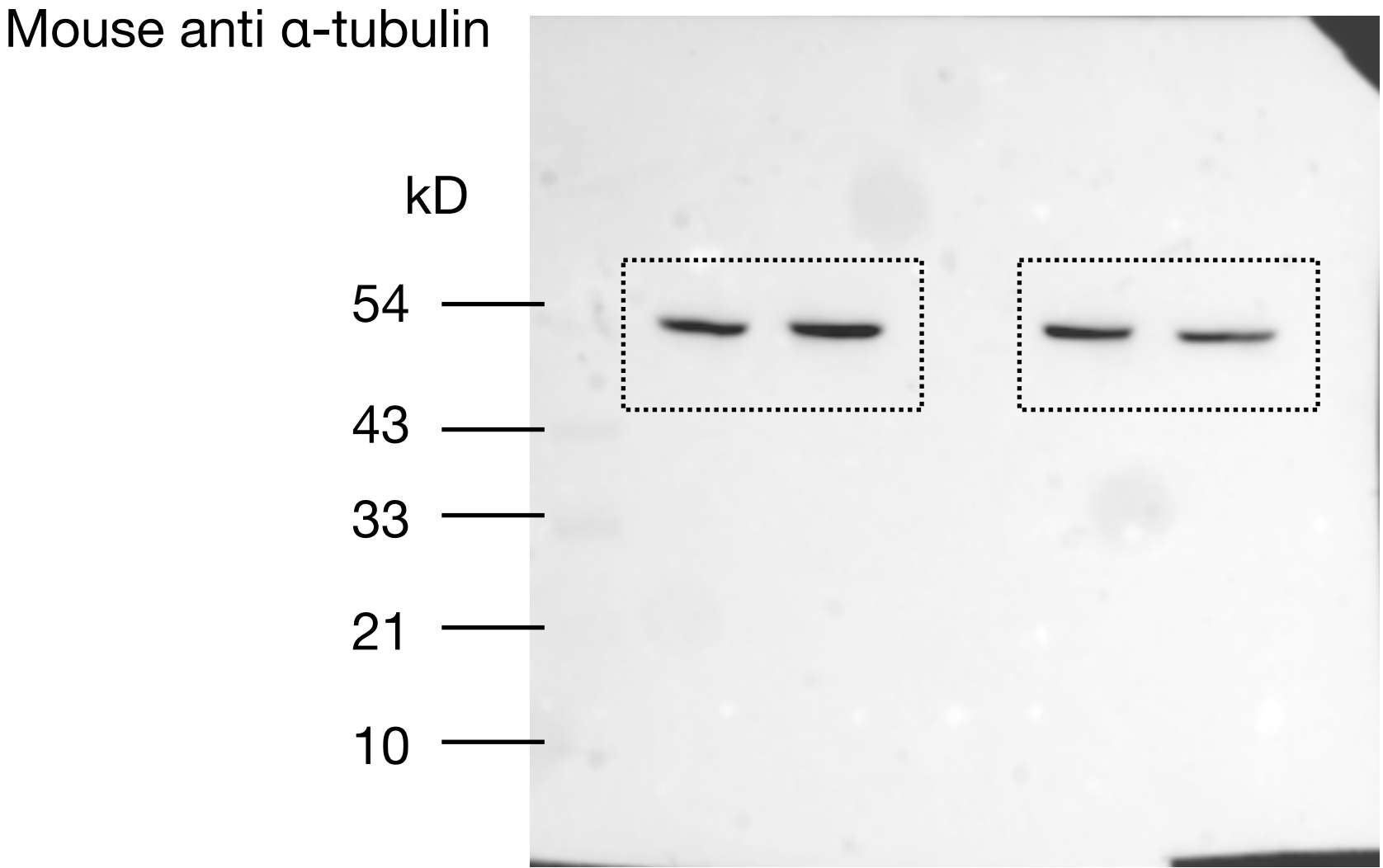

Fig S2C

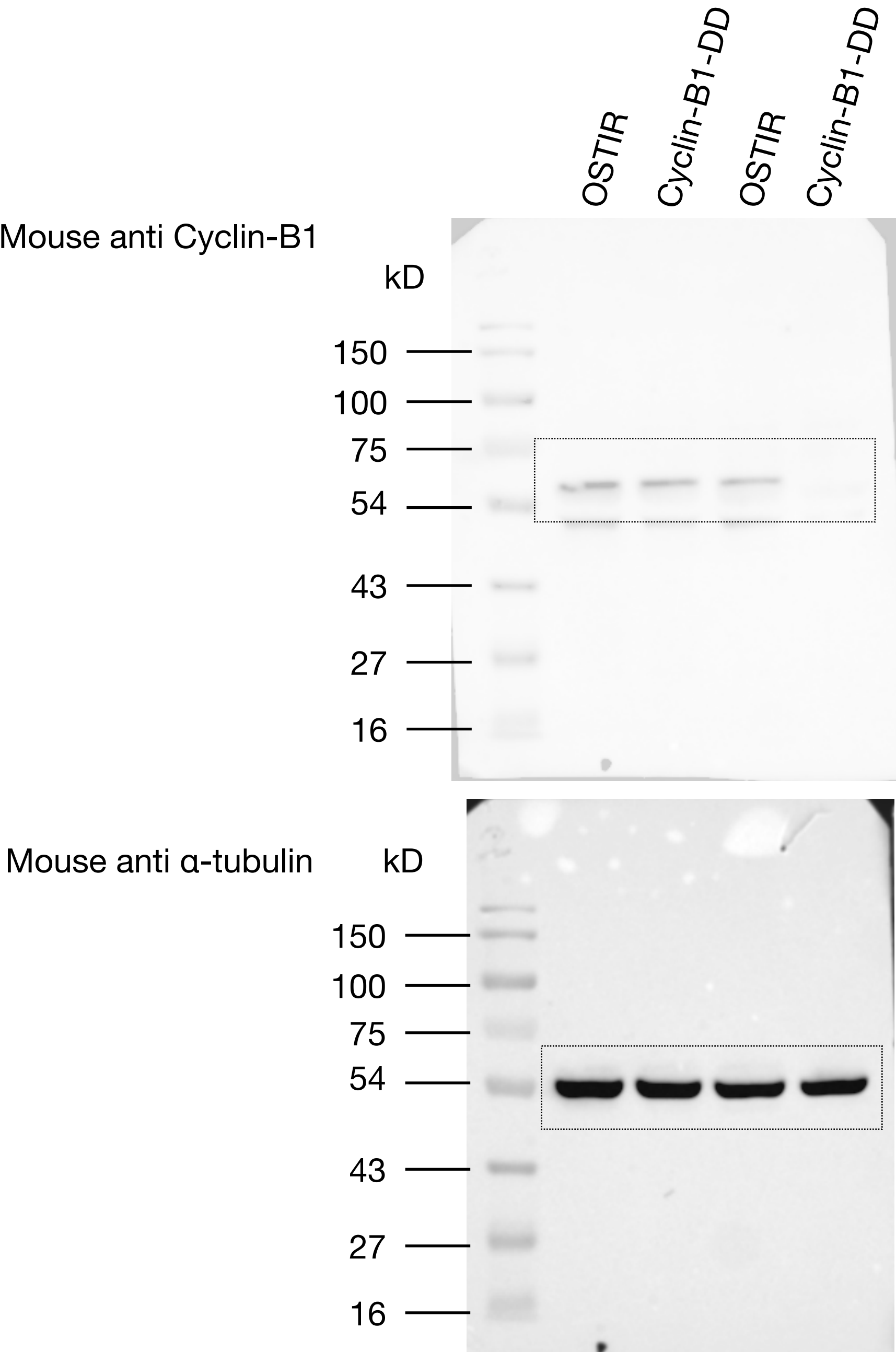

Fig S4D

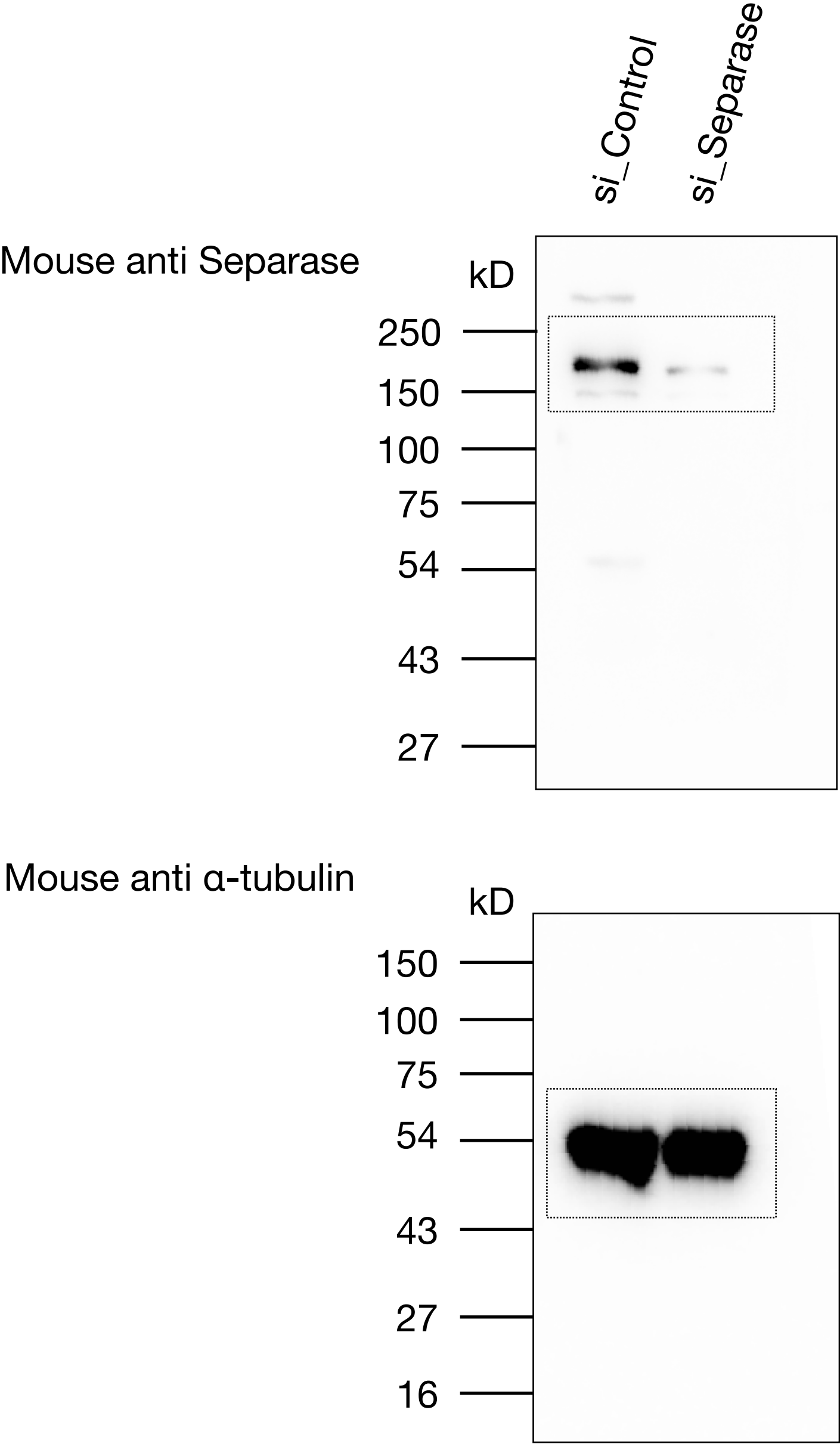

Supplement: Supplementary file 11 — Source Data [file 41467_2023_41753_MOESM11_ESM.zip › Source Data/Source Data Western Blots.pdf]
