# Supplementary material for: Mild replication stress causes premature centriole disengagement via a sub-critical Plk1 activity under the control of ATR-Chk1
Source: Nat Commun. 2023 Sep 29;14:6088. doi: 10.1038/s41467-023-41753-1 (PMC10541884; doi:10.1038/s41467-023-41753-1)
Supplement: Supplementary file 1 — Supplementary Information [file 41467_2023_41753_MOESM1_ESM.pdf]

Figure S1. Dwivedi et al.

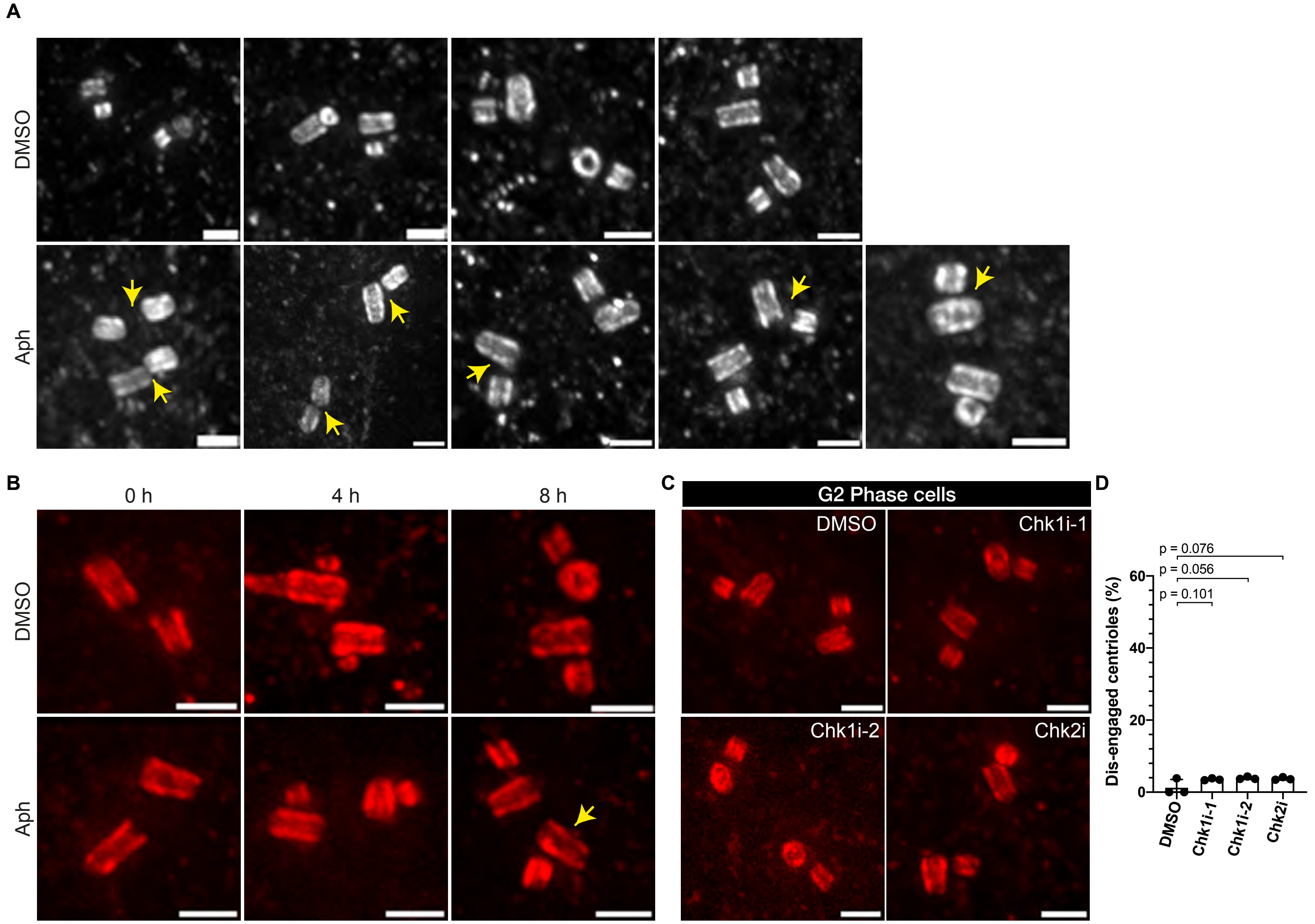

Figure S1: **Mild replication stress causes premature centriole disengagement in G2 and inhibition of Chk1 or Chk2 alone did not cause premature centriole disengagement**. (**A**) U-ExM images of centrioles in G2 phase RPE1 cells treated with indicated drugs and stained with α-tubulin. (**B**) U-ExM images of centrioles in RPE1 cells at indicated time after release from G1 arrest and stained against α-tubulin and treated with indicated drugs. (**C**) U-ExM images of centrioles, stained with α-tubulin, in G2 phase RPE1 cells treated with indicated drugs/inhibitors. (**D**) Quantification of percentage of G2 phase cells (shown in C) having disengaged centrioles within their centrosomes (N = 3 independent experiments, n = 78, 85, 77 and 81 cells in DMSO, Chk1i-1, Chk1i-2 and Chk2i, respectively) Data presented as Mean values ± SD. (p-values from two-tailed Sídak test). Scale bars = 0.5 µm. Source data for all graphs are provided as a Source Data file.

Figure S2. Dwivedi et al.

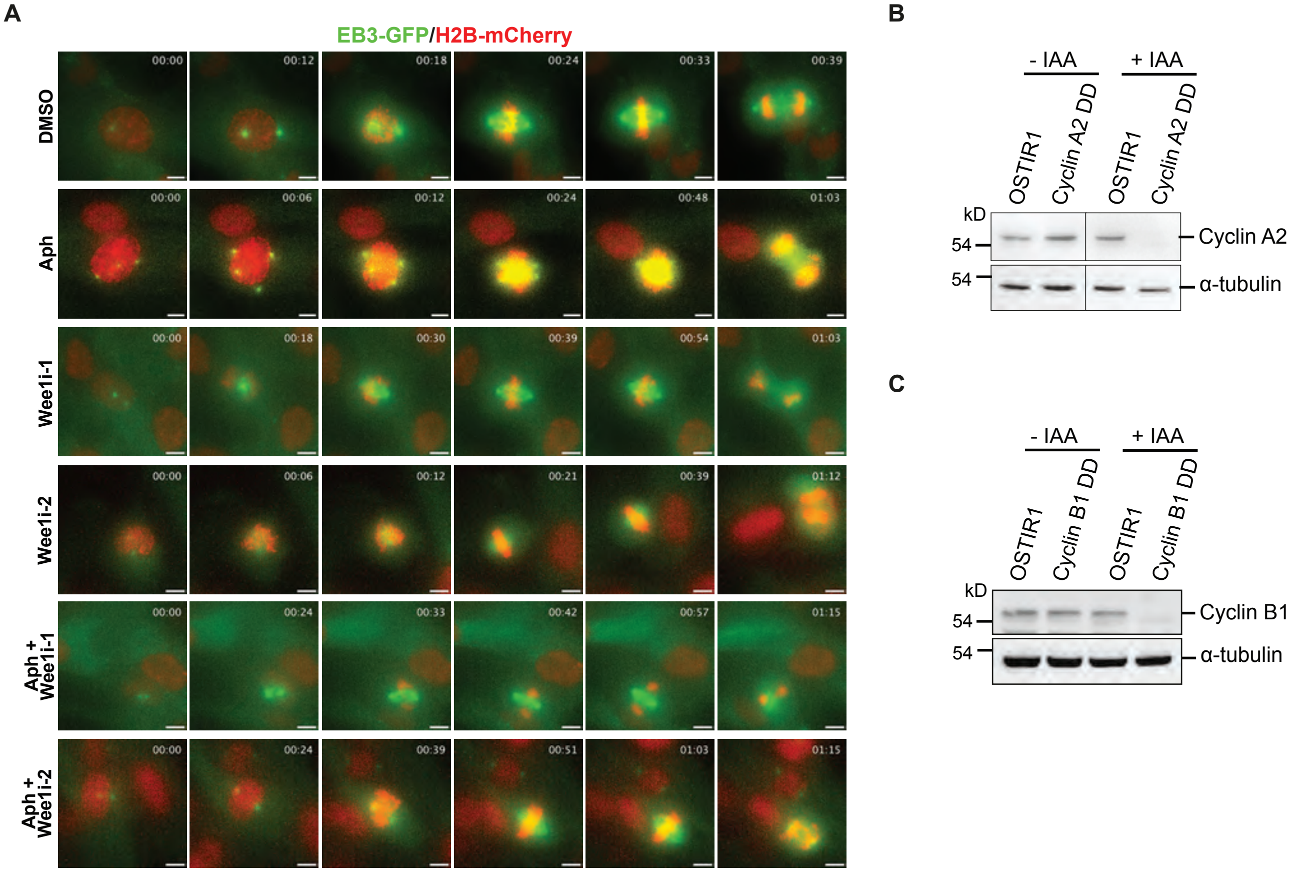

Figure S2: Wee1 activity in required for centriole disengagement and auxin treatment causes degradation of cyclins. (**A**) Live-cell time-lapse images (merged images) of RPE1: EB3-eGFP/H2B-mCherry cells treated with indicated drugs/inhibitors. Scale bars = 5 μm. (**B**) Western blot images showing degradation of Cyclin-A2 in Cyclin-A2 double degron containing RPE1 cells after addition of Auxin (IAA) but not in OSTIR Control RPE1 cells. The blots were also probed with α-tubulin to confirm equal protein loading. (**C**) Western blot images showing degradation of Cyclin-B1 in Cyclin-B1 double degron containing RPE1 cells after addition of Auxin (IAA) but not in OSTIR Control RPE1 cells. The blots were also probed with α-tubulin to confirm equal protein loading.

Figure S3. Dwivedi et al.

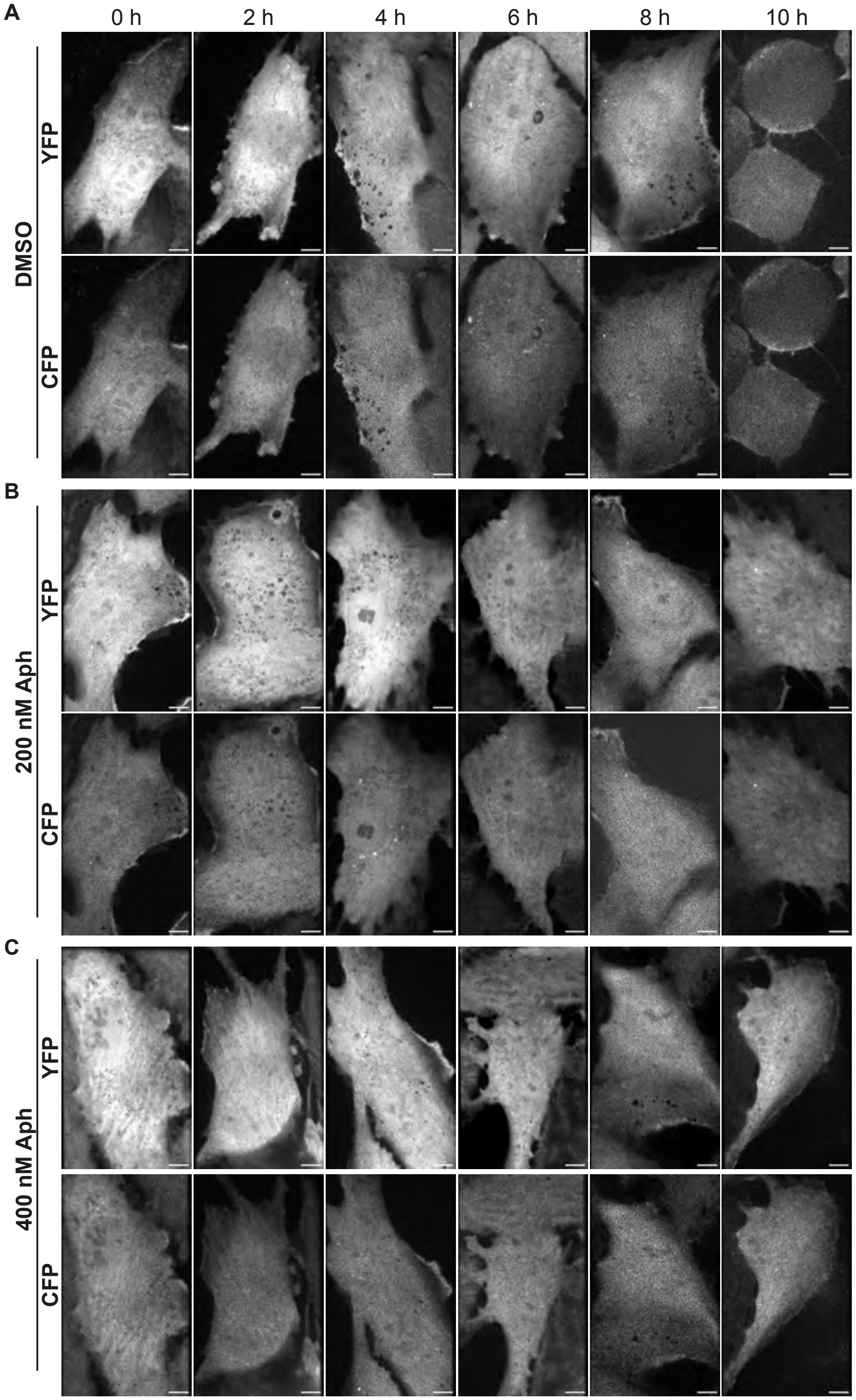

Figure S3: Mild Replication stress delays full Plk1 activation. (**A-C**) Representative images of CFP and YFP fluorescence at indicated time after Cdk4/6i release taken from RPE1 Plk1-FRET Sensor cells treated with indicated inhibitors/drugs.

Figure S4. Dwivedi et al.

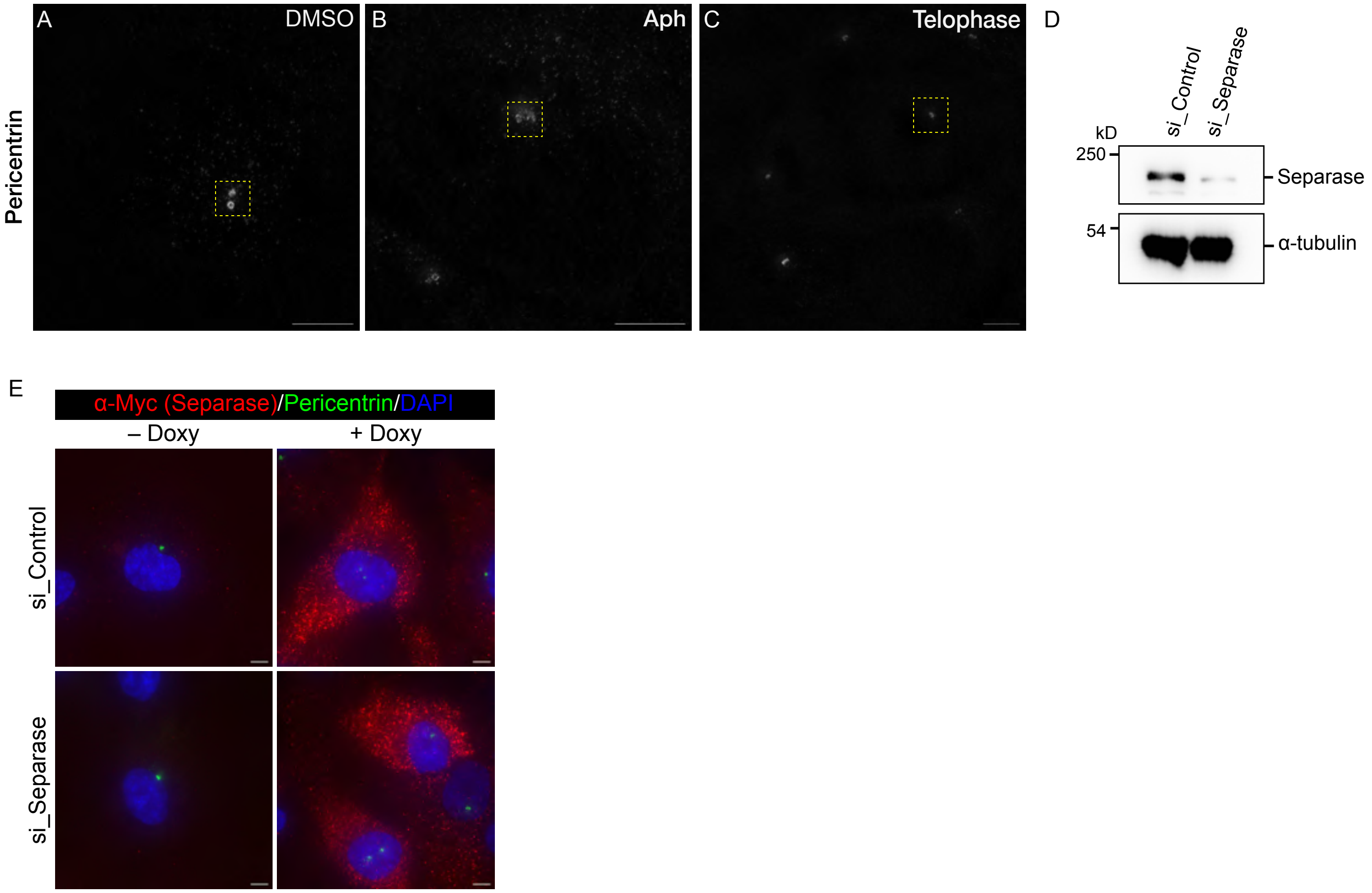

Figure S4: Replication Stress disrupts PCM integrity. (**A-C**) STED nanoscopy images of G2 phase hTERT-RPE1 cells treated either with DMSO (A) or Aph (B) and DMSO treated cells in telophase (C). The cells were stained for CENP-F for identification of G2 cells. Insets represent the pericentriolar region shown below and in Fig 7A (in grey). (**D**) Western blot images showing depletion of Separase in RPE1 cells after 72 h of siRNA transfection. The blots were also probed with α-tubulin to confirm equal protein loading. (**E**) Representative immunofluorescence images of RPE1:-Myc-Separase cells treated with indicated drugs and siRNAs and stained for Separase (α-Myc:red), Pericentrin (green) and DAPI (blue). Scale bars = 5 µm.
